# Compliance level and associated factors of iron folic acid supplementation among pregnant women in North Shoa Zone, Ethiopia

**DOI:** 10.1038/s41598-024-63111-x

**Published:** 2024-06-11

**Authors:** Daba Ejara, Amsal Ferede, Jemal Ebrahim Shifa, Fikadu Bekele, Tahir Ahmed Hassen

**Affiliations:** 1 School of Nursing, Mada Walabu University, Shashamene Campus, Shashamene, Ethiopia; 2 Wuchale Woreda Health Office, North Shoa Zonal Health Department, Muke Turi, Ethiopia; 3 School of Nursing and Midwifery, College of Health and Medical Sciences, Haramaya University, Dire Dawa, Ethiopia

**Keywords:** Compliance, Ethiopia, Iron folic acid, Pregnant women, Supplementation, Health care, Medical research

## Abstract

Iron deficiency is a widespread micronutrient deficiency, impacting over 30% of the global population. Iron Folic Acid supplement is recommended for pregnant women to counter iron deficiency anemia and neural tube anomalies. Although Iron Folic Acid supplementation is integral to Ethiopian antenatal care, one in four women in Ethiopia experiences anemia during pregnancy suggesting poor compliance. This study aimed to investigate compliance level and associated factors of Iron Folic Acid supplementation among pregnant women attending antenatal care in Wuchale Woreda of North Shoa Zone, Ethiopia. An institutional-based cross-sectional study was conducted among 302 pregnant women from March 20 to April 5, 2021, who were selected using a systematic random sampling technique. Data were collected through face-to-face interview, entered epi-data, and exported to Statistical Package for the Social Sciences for analysis. A multivariable logistic regression was used to identify factors associated with compliance level. All the results were presented with 95% confidence intervals. The compliance with Iron Folic Acid supplementation was 47.0%. Residing nearest to the health facility (AOR = 2.46; 95% CI 1.32, 4.57), initiating antenatal care at health center (AOR = 2.23; 95% CI 1.17, 4.51), having a family size of 4 and above (AOR = 4.99; 95% CI 2.43, 10.24), and receiving information from health extension workers (AOR = 5.52; 95% CI 1.30, 23.54) increased compliance with Iron Folic Acid supplementation. Less than half of the pregnant women were compliant with Iron Folic Acid utilization. There is a need to prioritize promoting the importance of Iron Folic Acid supplementation through health education particularly by targeting pregnant women with identified factors.

## Introduction

In order to sustain all normal cellular and molecular functions, our body needs to obtain essential micronutrients from the diet we consume^1^. Today, over two billion people worldwide suffer from dietary deficiencies of vitamins and minerals predominantly in developing countries, resulting in different adverse health outcomes. Nutritional status of a mother during pregnancy plays a vital role to prevent maternal morbidity and mortality and facilitate normal fetal development^1,2^.

Providing relatively high quantities of micronutrients as tablets, capsules, or syrups is known as supplementation. Iron and folic acid supplements are widely used by pregnant women to prevent and correct iron deficiency anemia and neural tube anomalies during gestation^3^.

Medication compliance is the act of following the health care provider's instructions for when to take a medication, how much to take, and how frequently to take it. Compliance is tracked over time and reported as a percentage^4,5^.

All pregnant women should begin receiving a standard 400 g dose of folic acid and 30–60 mg of iron as early in gestation as possible, according to World Health Organization (WHO) recommendations. Before giving birth, women should ideally consume 180 pills and should be continued for three months after giving birth. Accordingly, many countries recommend pregnant women to take 90 or more tablets throughout their pregnancy. A daily dose of 60 mg of elemental iron is preferred in areas where anemia in pregnant women is a severe public health problem (40%), along with 400 µg of folic acid. If the prevalence of anemia is 40% or more among pregnant women, supplementation should be continued for three months after delivery^4,6^.

Ethiopia has a national guideline for the prevention and treatment of anemia in pregnancy, as well as procedures for the distribution of Iron Folic Acid (IFA). As per this recommendation, all pregnant women should take 60 mg elemental iron (ferrous sulphate, ferrous fumarate, or ferrous gluconate) and 0.4 mg folic acid daily for six months, which is 180 tabs. The national nutrition Strategy and National guideline for preventing and controlling micronutrient deficiencies in Ethiopia emphasize the need for daily iron supplementation for at least six months during pregnancy^7,8^.

Supplementation of IFA tablets during pregnancy has been shown to lower the risk of maternal anemia and low birth weight^9^. An efficient distribution, infrastructure and high levels of customer compliance are required for IFA supplementation to produce better results^10^.

Even while many nations, including Ethiopia, have maintained antenatal care (ANC)-based IFA distribution programs for decades, they frequently have low coverage rates and don't fully realize their potential to reduce anemia^10^. Many managers of dietary supplement programs repeatedly indicate that low compliance and a lack of supply are the main obstacles to success^10,11^. Because of adverse effects and a general lack of enthusiasm, low therapeutic compliance, which is prevalent in underdeveloped nations, is preventing the supplements program from being successful^4,12^.

In many developing countries including Ethiopia, IFA supplementation is a crucial component of ANC and is given without charge. However, the consumer's compliance is essential to the success and effectiveness of such initiatives. According to several experts, non-compliance is one of the primary causes of national iron supplementation programs' failure^13,14^.

According to the Ethiopian Mini Demographic Health Survey 2019, 40% of pregnant women were not receiving any of the iron folic acid supplements. In addition, among those who had received, only 11% of them had taken the supplements for 90 days or more^15^. A systematic review and meta-analysis indicated that one in four women in Ethiopia experience anemia during pregnancy^16^. This could be partly related to pregnant women’s lack of awareness about anemia and its prevention methods including taking IFA supplements as per the recommendation.

In Ethiopia, some regional-based data indicates that only 2% of women who have at least one ANC visit were reported to receive at least one IFA tablet^17^, indicating unacceptable compliance rate among pregnant women. Although now well explored, factors such as socio-demographic characteristics, health system-related and patient-related factors are believed to be contributing to non-compliance with IFA supplementation during pregnancy^14,18,19^. Most importantly, due to variation among the study populations both within and between countries, the coverage of IFA compliance and driving factors for non-compliance may, sometimes, be population specific. This study aimed to investigate compliance level and associated factors of IFA supplementation among pregnant women attending ANC in North Shoa Zone, Ethiopia.

## Methods and materials

### Study design and setting

A cross-sectional, institutional-based study was conducted from March 20 to April 5, 2021 among pregnant women visiting ANC at government health facilities in Wuchale Woreda. Wuchale woreda, which is one of the 14 Woredas of North Shoa zone of Oromia Regional State, Ethiopia. The woreda is located 80 km away from the capital Addis Ababa, in the northern direction and it has 27 kebeles (small administrative units). As of 2021, the woreda contained an estimated 135,303 population with 4695 pregnant women. There are six health centers, 24 health posts, one primary hospital, six private clinics (2 medium and 4 primary), two pharmacy stores, and one rural drug vendor in the woreda.

### Population

The source population for this study consisted of pregnant women who had ANC in governmental health facilities in Wuchale Woreda. The study population included pregnant women who had previously taken IFA tablet as a supplement and who attended routine ANC services at the selected governmental health facilities. The individual study units were pregnant women who had received IFA supplements during their ANC follow-up at least one week prior to the survey and who presented at the health facilities. Pregnant women with serious illnesses were excluded from the study.

### Sample size determination

The required sample size was determined using the formula for single population proportion with finite source population correction. The assumptions were the proportion of compliance with IFA supplementation of 28.7%^20^, 95% confidence level and 5% margin of error. By considering 10% compensation for possible non-response, the final sample size was 307 pregnant women. The adequacy of the sample size for identifying selected factors for compliance level of IFA supplementation among pregnant women was also evaluated using post-hoc power analysis.

### Sampling technique

A lottery method was used to select four health centers (Wabari, Idoro, Ginbichu and Muka Turi) in Wuchale woreda. The registration books of each chosen health facility were used to compile a sample frame of 2468 mothers who were attending ANC. The study participants were selected by using a systematic random sampling technique based on the ANC registry (registration books), following the proportional allocation of the total sample size (n = 307) for the selected four health centers and an interval of k = N/n = 2468/307 = 8 was used to select the study participants. The first study participant was selected using the lottery method and then every 8th woman was included.

### Data collection

Data from pregnant women were gathered using a face-to-face interviewer-administered data collection technique. A structured questionnaire that had been pretested and translated into the local language (Afaan Oromo) was used to collect the data. Four trained health professionals who speak the same language with the study participants were recruited for data collection.

### Study variables

The dependent variable of the study was compliance with IFA supplementation categorized into two levels: compliance or non- compliance. There are no obvious clear-cut-offs points for IFA non-compliance. In this instance, IFA supplement compliance was assessed using the self-reported number of IFA pills consumed in the seven days prior to the survey as a proxy estimator for the recommended (90 days) of IFA compliance before the survey.

Socio-demographic characteristics of the pregnant mother; pregnancy and health status; knowledge on IFA supplementation; waiting time and counseling services during IFA supplementation, distance to health facilities, supply shortage, source of information and place where ANC was initiated; supply-related factors including duration, side effects, and taste of supplement were covariates considered for the study. Six questions about the advantages of IFA, its consumption frequency, duration, and side effects were used to gauge women's awareness of the supplement. Each item's correct response received a score of "1," while the wrong response received a score of "0."

### Operational definition

#### Compliance

Pregnant women who took at least five IFA pills per week—or at least 70% of the recommended dose—in the previous week before data collection were considered to be compliant with the supplement's recommendations^21^.

#### Adequate knowledge on anemia

The multiple-choice questions about the causes, symptoms, and prevention methods of anemia were used to assess knowledge of anemia. The correct answer was labeled as 1 and the wrong answer as 0. Items were summed up; the mean was calculated. A woman was considered to have adequate knowledge on anemia if she properly answered knowledge questions with a mean or above mean score.

#### Knowledge of iron-folate supplement

Knowledge of iron-folate supplement was assessed by summing up questions on the benefits of iron-folate supplementation and possible effects of iron deficiency anemia during pregnancy. The correct answer was labeled as 1 and the wrong answer as 0. Items were summed up; the mean was calculated. A woman was considered to have adequate knowledge on anemia if she properly answered knowledge questions with a mean or above mean score.

#### IFA supplementation

Combined tablet composed of 60 mg elemental iron and 0.4 mg folic acid that is given to pregnant women during pregnancy and postpartum^4^.

### Data management and analysis

Epi-data version 3.1 was used to enter the data, which was then exported to SPSS version 25 for analysis. To summarize the data, frequency distributions, tables, means, standard deviations, medians, minimums, and maximums of the study variables were employed. Binary logistic regression model was used to identify factors associated with compliance with IFA supplementation. All independent factors with p values less than 0.25 in the simple logistic regression model were chosen as candidate variables for the multivariable logistic regression. To identify independent factors associated with compliance with IFA supplementation among pregnant women, multiple logistic regression was carried out and OR along with 95% CI was used to measure the magnitude of association. Statistical significance of the association between outcome and explanatory variables was declared at p-value less than 0.05. Variance inflation factor was used to determine the absence of multicollinearity. Using the Hosmer and Lemeshow goodness-of-fit test statistic, the model fitness was checked prior to declaring the association.

### Ethics approval and consent to participate

The study was approved by the ethical review committee of Addis Ababa Medical and Business College with registration number AAMBC/stu/1925/13. Letter of permission was obtained from North Shoa Zone and Wuchale woreda health office. Prior to data collection, informed consent was obtained from pregnant mothers. All methods were carried out in accordance with relevant guidelines and regulations.

## Results

### Socio-demographic and economic characteristics of the study participants

Three hundred two pregnant women participated in this study, making a response rate of 98.4%. One hundred five (34.8%) of the respondents were between the age of 25–29 years. The mean (± SD) age of the participants was 28.5 (± 5.18) years. The majority, 275 (91.1%), of the study participants were married. Regarding educational status, 114 (37.7%) were unable to read and write and 61 (20.2%) had attended primary school. Slightly over two-thirds of the study participants were housewives while government employees accounted for 45(14.9%), by profession. Nearly half, 162 (53.6%) of the participants had an average monthly income of USD18.16–54.40 (Table 1).

**Table 1:** Socio-demographic and economic characteristics of pregnant women attending antenatal care in health facilities of Wuchale Woreda, Oromia Region, 2021.

Variables	Frequency	Percent
Age (years)		
≤ 24	61	20.2
25–29	105	34.8
30–34	90	29.8
≥ 35	46	15.2
Residence		
Urban	73	24.2
Rural	229	75.8
Educational status of the women		
Unable to read and write	114	37.7
Read and write	59	19.5
Primary	61	20.2
Secondary	31	10.3
Tertiary	37	12.3
Husband’s educational status		
Unable to read and write	107	36.6
Read and write	55	18.8
Primary	64	21.9
Secondary	28	9.6
Tertiary	38	13.0
Occupational status of the women		
Housewives	210	69.5
Government employee	45	14.9
Merchant	18	6.0
Daily laborer	29	9.6
Monthly income		
< USD18.14	63	20.9
USD18.16–54.40	162	53.6
> USD54.42	77	25.5
Family size		
≤ 3	124	41.1
≥ 4	178	58.9

### Pregnancy and health-related characteristics of the respondents

Majority of the study participants were multi-gravida. Two-hundred forty-two (80.1%) of the participants had initiated their first ANC after 16 weeks of gestation and nearly three-fourth of them, 221 (73.2%), began their ANC follow-up at health centers. One hundred seventy-three (57.3%) participants had resided more than 5 KMs away from the health facility. The vast majority (77.8%) of the study participants had travelled to the health facilities on their feet. Majority, 267 (88.4%) of the survey participants had no history of abortion (Table 2).

**Table 2:** Pregnancy and health characteristics of pregnant women attending antenatal care in health facilities of Wuchale District, Oromia Region, 2021.

Variables	Frequency	Percent
Gravidity		
Primi gravida	55	18.2
2–4	154	51.0
≥ 5	93	30.8
Number of ANC visits		
1	46	15.2
2–3	199	65.9
≥ 4	57	18.9
Facility where ANC follow-up started		
Health post	81	26.8
Health center	221	73.2
Distance to health facility		
< 5 km	129	42.7
≥5 km	173	57.3
Means of transportation		
On foot	235	77.8
Animal back	30	10.0
Car	37	12.2
Health problem during this pregnancy		
Yes	54	17.9
No	248	82.1
Parity N = 246		
1–2	121	49.2
3–4	76	30.9
≥ 5	49	19.9
Abortion		
Yes	35	11.6
No	267	88.4
Stillbirth		
Yes	17	5.6
No	285	94.4

### Knowledge on anemia and its prevention

Two hundred fifty-four of the participants (84.1%) did know anemia. Of the participants who did know about anemia in general, 248 (97.6%) responded that pregnant women are susceptible to anemia when asked about risk groups. Among the study participants, 235 (77.8%) responded that they know the causes of anemia. Of the participants who know the causes of anemia, only 83 (35.4%) know iron deficiency as a cause of anemia. Nearly two-thirds (78.1%) of the participants know that anemia is preventable. (Table 3).

**Table 3:** Knowledge on anemia and its prevention among pregnant women attending antenatal care in health facilities of Wuchale District, Oromia Region.

Variables	Frequency	Percent
Knows about anemia		
Yes	254	84.1
No	48	15.9
Pregnant women susceptible to anemia (n = 254)		
Yes	248	97.6
No	4	2.4
Source of information about anemia		
Health professionals	234	77.5
Health extension worker	35	11.6
Mass media	33	10.9
Know causes of anemia		
Yes	235	77.8
No	67	22.2
What are causes of anemia (235)		
Unbalanced diet	123	52.3
Iron deficiency	83	35.4
Lack of blood	13	5.5
Others	16	6.8
Knows sign and symptoms of anemia		
Yes	240	79.5
No	62	20.5
Anemia prevented		
Yes	236	78.1
No	66	21.9

Others: chronic infection (TB, HIV), Malaria, Hookworm, schistosomiasis infection.

### Compliance with IFA supplementation

In this study, 144 (47.7%) of the participants had collected more than 90 tablets per visit. The prevalence of compliance with IFA supplementation (the proportion of pregnant women who took > 70% of IFA tablets per week) was 47.0% (95% CI 43.1–51.2). Many of the participants responded that they took one tablet per day and close to half of the participants took the tablets for the duration of more than four months. Majority of the survey participants, 278 (92.1%) responded that they knew the side effects of iron folate (Table 4).

**Table 4:** Compliance with IFA supplementation among pregnant women attending antenatal care in health facilities of Wuchale Woreda, Oromia Region, 2021.

Variables	Frequency	Percent
How many tablets did you collect per visit		
1–30	92	30.5
31–60	19	6.3
61–90	33	10.9
> 90	144	47.7
I don’t know	14	4.6
How did you take your supplement		
One daily	288	95.7
Others*	14	4.3
Know side effect of iron		
Yes	278	92.1
No	24	7.9
Side effect of iron/folate (n = 278)		
Vomiting	118	42.4
Heart burn	132	47.5
Constipation	18	6.5
Others**	10	3.6
For how long did you take the supplement?		
One month	46	15.2
Two months	23	7.6
Three months	53	17.5
Four months	17	5.6
More than four months	146	48.3
I don’t know	17	5.6

Others*: weekly, when I think I am sick, I don’t know.

Others**: abdominal cramp, diarrhea.

### Benefits and risks of IFA

**Figure 1:**
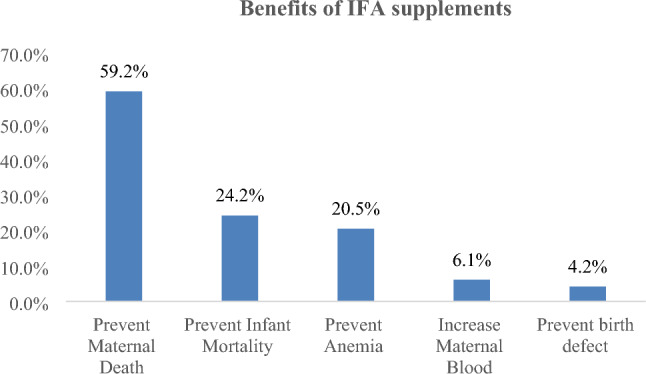
Knowledge on benefits of IFA supplementation among pregnant women attending antenatal care in health facilities of Wuchale Woreda, Oromia Region, 2021. *More than one answer was possible.

Majority of the respondents, 250 (82.8%) replied that IFA utilization has benefit. Among those who responded IFA has a benefit, 148 (59.2%) said that IFA prevents maternal death (Fig. 1).

### Health system and supply factors

Majority 265 (87.7%) of the respondents had counseling of which 181 (68.3%) counseled on the benefit of IFA supplementation (Table 5).

**Table 5:** Health system and supply factors among pregnant women utilizing IFA in Wuchale Woreda, Oromia Region, 2021.

Variables	Frequency	Percent
Counselled on IFA		
Yes	265	87.7
No	37	12.3
Counseling given on (n = 265, multiple answer is possible)		
Benefit of IFA supply	181	68.3
Frequency of supply	35	13.2
Side effect	62	23.4
Problem faced in the health facility		
Yes	85	28.1
No	217	71.9
What problem did you faced (n = 85)		
Shortage of supply	39	45.9
Long waiting time	25	29.4
Poor providers communication	21	24.7

### Factors associated with compliance with IFA utilization

The simple logistic regression analyses showed that compliance of IFA utilization had an association (*p* < 0.25) with maternal age, residence, educational status, occupation, family size, income, distance from health facility, knowing anemia, knowing causes of anemia, knowing iron, source of information, and place where ANC follow-up started.

In the multivariable analysis, after adjusting for the confounding factors, most of the variables that showed association in simple analysis were included. Among all variables entered in the multivariable logistic regression analysis, educational status, family size, place where ANC follow up started, distance from health facility and source of information were found to have statistically significant associated with compliance with IFA utilization.

Pregnant women who were unable to read and write were 4.42 times more likely to comply with IFA supplementation than those who had attended tertiary education (AOR = 4.42; 95% CI 1.32, 13.50). Similarly, pregnant women who had a family size of ≥ 4 were 4.99 times more likely to adhere to IFA supplementation compared to their counterparts with a family size of ≤ 3 (AOR = 4.99; 95% CI 2.43, 10.24). The odds of compliance with IFA supplementation were higher for those who started ANC follow-up at health centers than those who had follow-up at health posts. Pregnant women who started follow-up at a health center were 2.23 more likely to comply with IFA supplementation than those who started follow-up at the health post (AOR = 2.23; 95% CI 1.17, 4.51). Distance from their home to a health facility was also significantly associated with compliance with IFA supplementation, with pregnant women who had resided within less than 5kms radius from the health facilities were 2.46 times more likely to adhere to IFA supplementation compared to those who had resided more than 5kms away from the health facilities (AOR = 2.46; 95% CI 1.32, 4.57). Pregnant women who got information on IFA utilization from health extension workers were 5.52 times more likely to comply with IFA supplementation when compared to those who got information from mass media (AOR = 5.52; 95% CI 1.30, 23.54) (Table 6).

**Table 6:** Factors associated with compliance with IFA utilization among pregnant women in Wuchale Woreda, Oromia Region, 2021.

Variables	Compliance of iron/folate		COR (95% CI)	AOR (95% CI)
	No (%)	Yes (%)		
Age (years)				
≤ 24	27 (44.7)	34 (55.7)	1	1
25–29	62 (59.0)	43 (41.0)	1.82 (0.96, 3.44)	1.49 (0.61, 3.61)
30–34	45 (50.0)	45 (50.0)	1.26 (0.66, 2.42)	0.43 (0.15, 1.22)
≥ 35	26 (56.5)	20 (43.5)	1.64 (0.76, 3.54)	0.64 (0.21, 1.94)
Residence				
Urban	31 (42.5)	42 (57.5)	1	1
Rural	129 (56.3)	100 (56.3)	1.75 (1.03, 2.10)	1.35 (0.64, 2.86)
Educational status of the women				
Unable to read and write	92 (80.7)	22 (19.3)	6.13 (2.74, 13.71)	4.42 (1.32, 13.50)*
Read and write	23 (39.0)	36 (61.0)	1.94 (0.41, 2.17)	0.64 (0.19, 2.15)
Primary	24 (39.3)	37 (60.7)	0.95 (0.41, 2.19)	0.82 (0.24, 2.78)
Secondary	6 (19.4)	25 (80.6)	0.35 (0.12, 1.06)	0.28 (0.07,1.01)
Tertiary	15 (40.5)	22 (59.5)	1	1
Occupational status of the women				
Housewives	113 (53.8)	97 (46.2)	0.82 (0.37, 1.81)	1.22 (0.40, 3.74)
Government employee	20 (44.4)	25 (55.6)	0.57 (0.22, 1.45)	1.14 (0.27, 4.75)
Merchant	10 (55.6)	8 (44.4)	0.88 (0.27, 2.90)	2.05 (0.43, 9.81)
Daily laborer*	17 (58.6)	12 (41.4)	1	1
Monthly income				
< USD18.14	42 (66.7)	21 (33.3)	2.4 (1.20, 4.80)	2.23 (0.87, 5.73)
USD18.16–54.40	83 (51.2)	79 (48.8)	1.26 (0.73, 2.17)	1.58 (0.72, 3.46)
> USD54.42	35 (45.5)	42 (54.5)	1	1
Family size				
≤ 3	41 (33.1)	83 (66.9)	1	1
≥ 4	119 (66.9)	59 (33.1)	4.8 (2.51, 6.65)	4.99 (2.43, 10.24)***
Facility where ANC follow-up started				
Health post	36 (44.4)	45 (55.6)	0.63 (0.38, 1.05)	1
Health center	124 (56.1)	97 (43.9)	1	2.23 (1.17, 4.5)*
Distance health facility				
≥ 5 km	80 (46.2)	93 (53.8)	1	1
< 5 km	80 (62.0)	49 (38.0)	1.90 (1,19, 3.02)	2.46 (1.32, 4.57)**
Knows anemia				
Yes	129 (50.8)	125 (49.2)	1	1
No	31 (64.6)	17 (35.4)	1.77 (0.93, 3.35)	1.24 (0.49, 3.13)
Knows cause of anemia				
Yes	114 (48.5)	121 (51.5)	1	1
No	46 (68.7)	21 (31.3)	2.33 (1.31, 4.14)	1.29 (0.57, 2.91)
Knows drug called iron				
Yes	126 (49.6)	128 (50.4)	1	1
No	34 (70.8)	14 (29.2)	2.47 (1.26, 4.82)	1.95 (0.72, 5.29)
Source of information				
Mass media	18 (54.5)	15 (45.5)	1	1
Health professionals	112 (47.9)	122 (52.1)	0.77 (0.37, 1.59)	0.92 (0.32, 2.67)
Health extension workers	30 (85.7)	5 (14.3)	5.00 (1.55, 16.09)	5.52 (1.30, 23.54)*

^1^Reference.

*P-value < 0.05; **p-value < 0.01; ***p-value < 0.001.

## Discussion

This study assessed the Compliance level and associated factors of IFA supplementation among pregnant women attending ANC at government health facilities. The finding indicated that 47% of pregnant women complied with IFA supplementation during pregnancy. Factors such as distance from the health facility, family size, source of information about anemia, place where ANC started, and educational status were found to be independently associated with compliance with IFA supplementation among pregnant women.

The compliance level reported in our study was lower than the one reported in the earlier studies conducted in four major regions (Tigray, Amhara, Oromia and Southern Nations Nationalities and Peoples regions) of Ethiopia, Dire Dawa, and Dangila district of northern Ethiopia which reported the compliance level of 74.9%^14^, 71.8%^22^ and 79.6%^23^, respectively. This variation could be, in part, attributed to how the compliance level was measured including the timing and the cut-off points used. For example, while the study conducted in Dire Dawa^22^ and Dangila district^23^ measured the compliance level as consumption of four or more IFA tablets per week, our study considered consumption of at least five tablets per week in the week preceding the survey to define the compliance level, which might have contributed to the observed low compliance level. In addition, there might be differences in socio-demographic characteristics and level of community awareness about the importance of IFA supplementation.

Of note, the finding of the current study is also lower than those of some low-and middle-income countries such as Nigeria^24^, India^18^, and Nepal^25^, which reported the compliance level of 65.9%, 64.7%, and 55.7%, respectively. These disparities in compliance levels could stem from various factors, such as variations in healthcare infrastructure and other socio-economic factors. For instance, in the study conducted in Nepal^25^, only 32% of the participants had resided in rural areas and 46% had travelled less than or equal to 30 min to access health facilities, compared to our study, in which nearly 75% of the participants were from rural areas and 57% travelled more than five kilometers to access the nearest health facilities, likely contributing to the lower compliance level in our setting.

Yet, our finding is higher than the one reported in the previous studies that were conducted in the Mecha district of the Amhara region (20.4%)^10^, Misha district of south Ethiopia (39.2%)^21^, and Northwestern Zone of Tigray in Ethiopia (37.2%)^26^. This discrepancy might be attributed to the level of awarness of pregnant women about anemia and its prevention. For instance, in the study conducted in Misha ditrict^21^, only 56.1% of pregnant women had a good knowledge of anemia while in our study, 84.1% of pregnant women reported to know about anemia, likely increasing the compliance level in the current study. The observed variation in the awarness of pregnant mother could be attributed to the timing of the studies. Notably, the studies conducted in Mecha district of the Amhara region^10^ and Misha district of south Ethiopia^21^ were conducted in 2013 and 2015, respectively, as compared to our study which was conducted in 2021. This timeframe encapsulates several years during which various strategies aimed at enhancing IFA supplementation and raising awareness were implemented by the Ethiopian government as part of broader initiatives to improve maternal and child health outcomes. This enhanced awareness, in turn, potentially contributed to the relatively high compliance level in the current study.

Pregnant women who had resided within less than a five-kilometer distance from the health facility were found to be more complied with IFA supplementation compared to those who resided more than or equal five kilometers away from the health facilities. This is supported by the findings from the previous studies, Dangila study,^23^ and North-West Tanzania^27^ that reported a higher level of compliance among women who resided within a short distance from the nearest health facility. Pregnant women who live in closer proximity to health facilities are more likely to have easier access to healthcare services. As a result, these women are better positioned to engage with healthcare interventions, including adhering to recommended IFA supplementation.

Pregnant women who were unable to read and write were approximately four times more likely to comply with IFA supplementation than those who had attended tertiary education, contradicting with some of the previously documented literature^12,20,25,28^ which indicated positive association between education and higher compliance with IFA supplementation. Our finding, however, should be interpreted with caution as there might be some unmeasured confounders. One plausible unmeasured confounder could be the interplay between health literacy and education. Women with lower education levels might place greater reliance on healthcare providers' guidance due to limited health literacy, thus fostering adherence to IFA supplementation recommendations. Conversely, women with higher levels of education could potentially possess the capabilities and resources necessary to autonomously seek out information and make decisions concerning health^29^ which could consequently contribute to divergent patterns of adherence. Furthermore, social support networks could also be unmeasured variables impacting both education and compliance.

This study also found that family size was associated with compliance with IFA supplementation. Pregnant women with a family size of more than four were approximately five times more likely to comply with IFA utilization than those with a family size less than three. This is supported by the findings reported in the study conducted in Lay Armachiho health centers, Northwest, Ethiopia^20^. The possible reason could be due to experience from previous pregnancies as those pregnant women might have had opportunities to meet with health professionals and receive counseling during their previous pregnancies.

The findings from this study also highlighted that source of information was associated with compliance with IFA supplementation. Pregnant women who got information from health extension workers were 5.52 times more likely to adhere to IFA utilization when compared to those who got information from mass media. This might be due to the accessibility of information from health extension workers and low media coverage targeting the importance of IFA supplementation, especially at the rural level of the woreda.

Our findings also showed that pregnant women who started ANC follow-up at health centers were more likely to comply with IFA than those who started at health posts. In Ethiopia, health posts are at the bottom of the primary health care hierarchy and are staffed by two health extension workers, whereas health centers are the next level up and have more staff in terms of number, professional qualification, and set-up. As a result, the difference might be attributed to the more effective counseling skills of ANC providers at health centers.

## Limitation

The findings of the study should be interpreted in consideration of the following limitations. The study was conducted among pregnant women who attended their ANC follow-up in public health institutions; hence these findings might not be generalizable to mothers who attended their ANC at private health facilities as differences in socioeconomic characteristics are likely. Desirability bias may also be introduced as women who didn’t take the tablet may simply report as it was taken.

## Conclusion

The findings of this study indicated that more than half of the pregnant women in the study area still didn’t comply with IFA utilization, which in turn, implies that many women were at risk of iron deficiency anemia and neural tube defect. The importance of IFA utilization both for the health of the mother and the fetus was recognized by a large majority of respondents, but compliance to use still remains relatively low in the study area. Promoting the importance of IFA supplementation through health education and addressing the identified driving factors are needed to improve the uptake of IFA during pregnancy.

## Data Availability

All data and materials are available. The datasets used or analyzed during the current study are available from the corresponding author upon reasonable request.

## Abbreviations

ANC: Antenatal care
AOR: Adjusted odds ratio
COR: Crude odds ratio
HIV: Human immune virus
IFA: Iron folic acid
mg: Milligram
µg: Microgram
SPSS: Statistical package for social sciences
